# PRMT3 at the crossroads of inflammation: dual roles in metabolic reprogramming and immune dysregulation in chronic diseases

**DOI:** 10.3389/fimmu.2025.1663258

**Published:** 2026-01-09

**Authors:** Huining Hou, Jiaojiao Meng, Yingqing Chen, Xili Zhang, Zhuqing Yang, Qianqian Wang, Min Zheng, Zebo Jiang

**Affiliations:** 1 Translational Medicine Research Center, Medical School, Dalian University, Dalian, Liaoning, China; 2 Pharmacy Department, Jinan Maternity and Child Care Hospital, Jinan, Shandong, China; 3 Department of Neurology, Affiliated Hospital of Yanbian University, Yanji, Jilin, China; 4 Zhuhai Hospital of Integrated Traditional Chinese and Western Medicine, Zhuhai, Guangdong, China

**Keywords:** protein arginine methyltransferase 3, chronic inflammatory diseases, asymmetric dimethylarginine, SGC707 inhibitor, metabolic reprogramming

## Abstract

Protein arginine methyltransferase 3, a type I PRMT family member, plays pleiotropic roles in chronic inflammatory diseases through its catalysis of asymmetric dimethylarginine modifications. Chronic inflammation, marked by metabolic dysregulation, immune dysfunction, and tissue fibrosis, drives diverse pathologies including non-alcoholic fatty liver disease, chronic kidney disease, and atherosclerosis. In this review, we comprehensively dissect the multifaceted contributions and molecular mechanisms of PRMT3 in inflammation-associated disorders. Mechanistically, PRMT3 aggravates inflammatory-metabolic dyshomeostasis in chronic inflammation via LXRα/HIF-1α methylation, thereby accelerating vascular calcification and fibrosis. Paradoxically, it simultaneously suppresses antiviral immunity and facilitates tumor immune evasion, underscoring its dual role as a molecular “double-edged sword”. Notably, PRMT3 inhibitors such as SGC707 demonstrate preclinical promise in modulating lipid metabolism and curtailing tumor progression. However, challenges persist regarding tissue specificity and off-target toxicity, necessitating further refinement. Collectively, these results provide a new molecular basis for therapeutic approaches targeting PRMT3.

## 1 Introduction

Post-translational modifications of proteins have gained significant attention in recent years owing to their critical roles in maintaining cellular functions and diversifying the proteome. Among post-translational modifications, arginine methylation serves as a key regulatory mechanism, driven by the dynamic interplay between protein arginine methyltransferases (PRMTs) and demethylases (1). To date, nine mammalian PRMT genes have been identified, classified into three subtypes based on their methylarginine products. Type I enzymes (PRMT1, 2, 3, 4, 6, and 8) catalyze monomethylarginine (MMA) intermediates, which are further converted into asymmetric dimethylarginine (ADMA). The type II enzymes (PRMT5 and PRMT9) catalyze the formation of MMA intermediates, which are subsequently converted into symmetric dimethylarginine (SDMA). As a pivotal member of the type II PRMT family, PRMT5 has been extensively studied for its functional roles. Our previous investigations (2, 3) demonstrated that PRMT5 plays a critical role in tumorigenesis and epigenetic regulation by mediating symmetric dimethylation of substrate proteins. In contrast, type III enzymes including PRMT7 exclusively produce MMA without proceeding to demethylation (4). PRMT1, the first PRMT family member discovered in 1996, remains the most extensively studied. PRMT3 was initially identified via a yeast two-hybrid screen using PRMT1 as bait and has since been implicated in ribosome biogenesis through dimethylation of the 40S ribosomal protein S2. Despite its predominant cytoplasmic localization, PRMT3 methylates histone H4 peptides *in vitro (*5, 6). Structurally, PRMT3 harbors a conserved catalytic core domain common to PRMTs and a unique N-terminal domain. In mammals, PRMT3 exhibits distinct functional characteristics compared to other family members, primarily mediating arginine methylation across diverse substrates to regulate cellular processes.

PRMT3 modulates biological processes such as gene expression, signal transduction, and metabolic reprogramming by catalyzing ADMA of arginine residues. Inflammatory diseases, including non-alcoholic fatty liver disease (NAFLD), atherosclerosis, chronic kidney disease (CKD), and chronic viral hepatitis are characterized by metabolic dysfunction, immune activation, and tissue fibrosis. Recent evidence highlights PRMT3’s significant involvement in inflammation, particularly in how it alters the methylation status of key molecules and modulates various inflammatory pathways and metabolic processes.

PRMT3 may drive inflammatory progression by altering the methylation status of key molecules, including hypoxia-inducible factor 1-alpha (HIF-1α), liver X receptor α (LXRα), and ADMA, thereby modulating critical inflammatory pathways such as the cGAS-STING and NF-κB signaling cascades, as well as metabolic processes like lipid synthesis and glycolysis. Recent research shows that a high-fat environment strongly increases PRMT3 expression. PRMT3 binds directly to the ligand-binding domain of LXRα, enhancing its transcriptional activity and driving overexpression of key lipogenic genes like sterol regulatory element-binding protein 1c, fatty acid synthase, and acetyl-CoA carboxylase. This mechanism drives abnormal activation of hepatic triglyceride biosynthesis, accelerating NAFLD progression (7). PRMT3 also plays a role in vascular complications associated with CKD. Higher PRMT3 levels correlate with vascular medial calcification in CKD patients. Mechanically, PRMT3 modifies HIF-1α via arginine methylation, stabilizing the protein by blocking its ubiquitination and degradation. Activated HIF-1α triggers glycolytic reprogramming, increasing glucose transporter 1, pyruvate kinase M2, and lactate dehydrogenase A (LDHA) expression (8). This metabolic shift drives vascular smooth muscle cells (VSMCs) to transform into osteoblast-like cells, causing vascular calcification and stiffer arteries, which raise cardiovascular risk.

However, PRMT3’s role in inflammation remains contentious. For instance, its anti-inflammatory activity in antiviral immunity contrasts with its pro-inflammatory effects in microenvironmental regulation, and its interactions with other PRMT family members remain poorly understood. This review synthesizes current knowledge on PRMT3’s molecular mechanisms in metabolic reprogramming, immune regulation, and tissue injury, evaluates its potential links to inflammatory diseases, addresses existing controversies, and identifies research gaps and future directions.

## 2 Structural features and catalytic mechanisms of PRMT3

**Figure 1:**
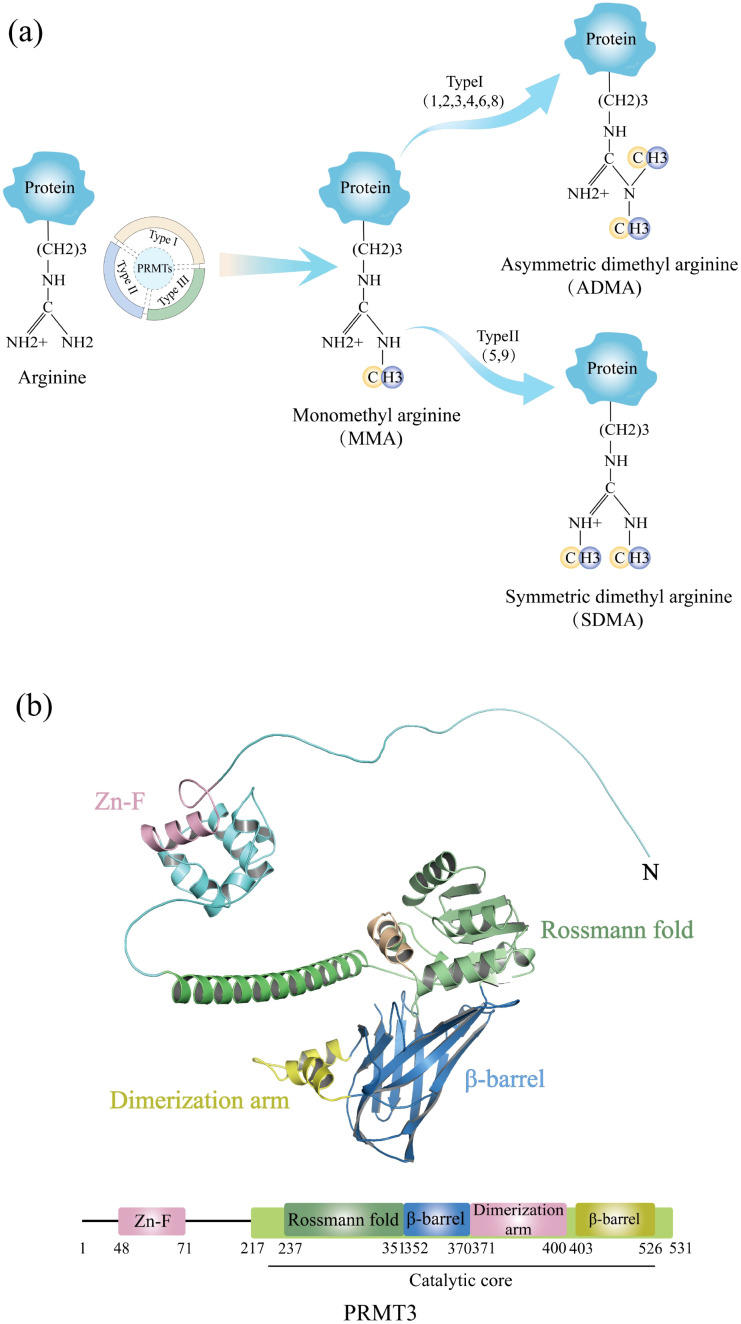
**(a)** Schematic diagram of arginine and its methylated products catalyzed by PRMT3. **(b)** Schematic diagram of the multi-domain architecture of PRMT3. The figure depicts the structural domains and key structural elements of PRMT3.

PRMT3, encoded by a 12.5-kilobase gene on human chromosome 11, is widely expressed in human tissues and catalyzes arginine monomethylation and subsequent asymmetric dimethylation. Its catalytic activity relies on a multi-domain architecture comprising conserved structural motifs that coordinate substrate recognition and methyltransferase function (**Figure 1**).

### 2.1 Catalytic core, AdoMet binding domain, and structural features

PRMT3 contains two functionally and structurally distinct domains critical for its enzymatic activity. The C-terminal catalytic domain adopts a conserved barrel-shaped conformation, with its active site formed by three helices at the subdomain interface. Key catalytic components include a double E-loop formed by Glu338 and Glu339 and a His254/Asp256 proton relay system, which collectively mediate the methyl transfer from S-adenosylmethionine (AdoMet) to substrates, a mechanism shared among PRMTs. However, PRMT3 is distinguished by a unique three-helix arm that enhances substrate selectivity. The N-terminal domain contains a modified Rossmann fold stabilized by αX and αY helices. This structural motif binds to AdoMet, facilitating methyl group transfer in PRMT3 catalysis. Unlike other PRMT family members, PRMT3 incorporates a C_2_H_2_-type zinc finger motif (Cys40-Cys43-His111-His115) adjacent to this domain. This motif likely fine-tunes substrate recognition and facilitates interactions with RNA or RNA-binding proteins, suggesting a specialized role for PRMT3 in RNA-related methylation processes. The synergistic combination of the catalytic core, the AdoMet-binding domain and the zinc finger motif reflects the structural uniqueness and functional plasticity of PRMT3, highlighting its ability to mediate the specific methylation of a wide range of substrates.

Furthermore, the zinc finger domain acts as the core module for PRMT3 to recognize natural substrates. It maintains an active conformation via zinc ion coordination through Cys40-Cys43-His111-His115. Without this zinc finger domain, PRMT3 can still catalyze the methylation of the artificial substrate GST-GAR but loses all ability to bind natural substrates, directly confirming this domain’s necessity for natural substrate recognition (9). In addition, differences in cofactors within the tissue microenvironment further regulate the zinc finger’s substrate preference. In the kidneys, high zinc ion concentrations stabilize the zinc finger conformation, facilitating PRMT3 binding to ADMA precursor proteins, which in turn enhances ADMA production. In the pancreatic cancer microenvironment, lactic acid functions as an allosteric modulator, altering the zinc finger-substrate binding interface and thereby increasing the methylation efficiency of lactate dehydrogenase A (10). In macrophages from recurrent miscarriage patients, macrophage migration inhibitory factor may regulate zinc finger activity through indirect effects, influencing PRMT3’s selection of arginine metabolism substrates (11).

### 2.2 Dynamic dimerization and enzymatic regulation

PRMT3 forms functional dimers via hydrophobic interactions between the three-helix arm of one protomer and the AdoMet binding domain of another. Full-length PRMT3 exists in a monomer-dimer equilibrium, where dimerization enhances enzymatic activity through three distinct mechanisms, stabilizing AdoMet binding improving substrate accessibility to the active site and facilitating interactions with large protein complexes such as chromatin remodeling complexes. PRMT3’s structural features provide critical opportunities for drug targeting, including the zinc finger motif as an allosteric site for inhibitor design, acidic residues in the E-loop as potential targets for competitive inhibitors and dimerization disruption to selectively inhibit enzymatic activity. These insights elucidate PRMT3’s catalytic roles. They also inform therapeutic strategies for PRMT3-associated diseases, such as cancer and metabolic disorders.

Beyond dynamic dimerization, post-translational modifications further impact PRMT3’s substrate contact efficiency by regulating its subcellular localization and protein stability. For phosphorylation, in pancreatic β cells, mycophenolic acid induces PRMT3 binding to RhoGDI-α while triggering Ser/Thr phosphorylation of PRMT3 (12). **T**his modification enhances PRMT3’s localization to the cytoplasmic membrane, allowing it to prioritize access to membrane-associated apoptotic substrates. In glioblastoma, Lys acetylation of PRMT3 promotes nuclear translocation, leading to co-localization with nuclear HIF-1α and thus prioritizing HIF-1α methylation (13). For ubiquitination, in acute leukemia PRMT3 degraders recruit E3 ubiquitin ligases to promote K48-linked ubiquitination and degradation of PRMT3. In contrast, p53 mutations in solid tumors reduce MDM2 expression, resulting in decreased ubiquitination and an extended half-life of PRMT3, thus providing a temporal window for its continuous modification of oncogenic substrates (14).

## 3 Diverse cellular functions and regulatory networks of PRMT3

The tissue-specific functional differences of PRMT3 stem primarily from interacting proteins specifically expressed in distinct tissues. These proteins confine PRMT3’s substrate pool through direct binding, enabling it to prioritize the modification of specific substrates. In tumor tissues, PRMT3 mainly interacts with oncogenic signaling molecules. In liver cancer, PRMT3 directly binds to IGF2BP1 and methylates its Arg501 residue, enhancing IGF2BP1’s stability toward target mRNAs and promoting oxaliplatin resistance (15). In colorectal cancer, PRMT3 binds to HIF-1α and methylates its Arg803 residue, inhibiting HIF-1α’s ubiquitin-mediated degradation and activating glycolytic genes (16). In endometrial cancer, PRMT3 interacts with the Arg418 residue of METTL14 and enhances METTL14’s m6A catalytic activity via methylation, thereby regulating ferroptosis-related genes (17). These oncogenic interacting proteins are exclusively expressed at high levels in tumors, confining PRMT3’s substrate pool to pro-tumor substrates.

In metabolic organs, however, PRMT3’s interacting proteins shift to metabolic regulatory molecules. In the liver, PRMT3 directly binds to the ligand-binding domain of LXRα, preventing LXRα from forming a heterodimer with RXRα and downregulating lipogenic genes such as SREBP-1c and FASN. In the kidneys, PRMT3 lacks cancer-associated interacting proteins and thus prioritizes binding to ADMA precursor proteins, generating ADMA through methylation and inhibiting NOS activity to mitigate fibrotic damage (18). This difference in interacting proteins is the primary driver of functional divergence between metabolic tissues and tumors.

PRMT3 regulates cellular processes through arginine methylation, including ribosome biogenesis, RNA metabolism, and cell fate control. By methylating the 40S ribosomal protein S2, PRMT3 maintains ribosomal subunit balance. Its deficiency disrupts the 40S:60S ratio without affecting precursor rRNA processing, indicating its role in late-stage ribosome maturation (19). PRMT3’s zinc finger domain facilitates interactions with RNA-associated substrates, influencing RNA processing. Unlike PRMT1, PRMT3 exhibits distinct substrate specificity, such as methylating GST-GAR and Yeast rmt1 Mutants. PRMT3 is critical for neural development with its loss impairing dendritic spine maturation and synaptic plasticity in rats (20). In bone metabolism, PRMT3 modulates miR-3648 during osteogenic differentiation, thereby regulating bone homeostasis (21). PRMT3 dysfunction contributes to disease pathogenesis. In cancer, PRMT3 drives glycolysis by methylating LDHA and HIF1α, promoting tumor growth and immune evasion. Ribosome assembly defects may activate nucleolar stress, inducing p53-dependent apoptosis. PRMT3 may also regulate transcription via chromatin remodeling, though further validation is required.

## 4 From metabolic imbalance to immune dysregulation: PRMT3 in chronic inflammation

Inflammation is a normal response of the immune system to infection or injury, playing a crucial role in maintaining homeostasis. Immune cells migrate to sites of injury or infection, where they interact with resident cells through immune mediators, thereby triggering appropriate immune responses. Generally, inflammation is beneficial for the body. However, when it occurs in healthy tissues or becomes chronic due to prolonged activation, it can lead to harmful effects. For instance, in the pathogenesis of sarcopenia, chronic inflammation promotes excessive muscle protein catabolism by activating the ubiquitin-proteasome system and the autophagic-lysosomal pathway (22). This process not only causes progressive muscle weakness and motor dysfunction in older adults but also creates a vicious cycle involving neurodegenerative diseases such as dementia, bone disorders like osteoporosis, and cancer cachexia (23). In cardiovascular pathology, chronic inflammation drives atherosclerotic plaque progression through oxidized low-density lipoprotein deposition, monocyte infiltration, and foam cell formation (24, 25). Notably, chronic inflammation affects the auditory system via the NF-κB/IL-6 signaling axis in two ways. Firstly, it induces apoptosis in cochlear spiral ganglion cells. Secondly, it exacerbates mitochondrial dysfunction within hair cells by elevating reactive oxygen species ROS levels, ultimately contributing to the development of presbycusis (26). Moreover, chronic inflammation drives tumor development through three core mechanisms. DNA damage, activation of signaling pathways, and remodeling of the immune microenvironment (27–29).

Numerous factors and cellular signaling pathways govern the development and progression of inflammation, including NF-κB, ROS, and noncoding RNAs. NF-κB is recognized as a central regulator of inflammation, modulating the expression of inflammatory cytokines and chemokines (30). Additionally, noncoding RNAs influence antigen-specific responses and immune cell differentiation by targeting specific substrates during chronic inflammation.

Recent research has revealed breakthrough insights into the multi-level regulatory functions of PRMT3. This epigenetic modifier serves as a critical molecular gatekeeper in antiviral immune responses and acts as a dynamic balancer in maintaining pregnancy immune tolerance. Zhu et al. (31) developed a zebrafish viral infection model, first showing that PRMT3 negatively regulates antiviral immunity. Mechanistic studies found PRMT3 expression increases 3.2-fold within 2 hours of viral infection, thereby suppressing virus-induced antiviral gene expression and interferon activation. The specific inhibitor SGC707 fully reverses this immunosuppression, indicating PRMT3’s role in innate immune regulation. Additionally, about 50% of recurrent miscarriage (RM) cases have unknown causes, with dysfunction of maternal-fetal interface immune cells and trophoblasts identified as key factors. Although NO prevents trophoblast apoptosis, its molecular mechanisms in RM remain unclear. ADMA, an endogenous nitric oxide synthase inhibitor generated by PRMTs, may regulate NO synthesis. Recent findings show excessive PRMT3 expression in decidual macrophages promotes RM by increasing ADMA levels, reducing NO production, and inducing trophoblast apoptosis. The PRMT3/ADMA/NO signaling axis thus represents a potential new target for RM diagnosis and treatment.

Chronic inflammatory diseases (CIDs) comprise a diverse group of disorders marked by persistent low-grade inflammation and immune dysfunction, affecting multiple organ systems such as the respiratory, gastrointestinal, and cardiovascular systems (11). Clinically, CIDs present as prolonged inflammatory states lasting months to years, including conditions like chronic obstructive pulmonary disease, asthma, inflammatory bowel disease, atherosclerosis, and rheumatoid arthritis (32–34). In industrialized nations, CIDs account for over 60% of mortality, with rising incidence linked to environmental triggers and aging populations, thus posing a significant challenge to global public health systems. The pathogenesis of CIDs hinges on three interconnected axes.

The first axis is the immune dysregulation and signaling pathway abnormalities. Sustained activation of pro-inflammatory cytokines drives hyperactivation of classical signaling cascades, including NF-κB, JAK/STAT, and the NLRP3 inflammasome pathway. This establishes a self-sustaining cycle of tissue damage and impaired repair. An imbalance in macrophage polarization characterized by the dominance of pro-inflammatory M1 phenotypes, combined with T-cell exhaustion and oxidative stress marked by the accumulation of reactive oxygen and nitrogen species ROS and RNS, collectively sustain a chronic inflammatory microenvironment. Then is the metabolic reprogramming and vascular remodeling. Inflammatory cells in diseased tissues undergo metabolic shifts, notably glycolytic and lipid metabolism rewiring driven by HIF-1α. These adaptations meet the energy demands of hyperactive immune cells while promoting angiogenesis and immunosuppressive microenvironments. For example, continuous vascular endothelial growth factor secretion in tumor-associated inflammation facilitates pathological new blood vessel formation and tumor immune evasion. And thirdly, it is the bidirectional association with carcinogenesis. Chronic inflammation promotes cancer development through genotoxic stress mediated by DNA damage induced by ROS, constitutive activation of pro-survival pathways such as the NF-κB signaling cascade, and disruption of immune surveillance. Classic examples include hepatitis-induced hepatocellular carcinoma and colitis-associated colorectal cancer. Conversely, tumor-derived inflammatory mediators further enhance immunosuppression, forming a feed-forward loop that worsens both malignancy and systemic inflammation (35).

Chronic inflammatory diseases involve a metabolic-immune crosstalk loop, with PRMT3 emerging as a critical modulator. As a class I arginine methyltransferase, PRMT3 mediates ADMA modifications to orchestrate dual roles in metabolism, immunity, and tissue repair (7, 21). At the metabolic level, PRMT3 exhibits dual regulatory functions with spatiotemporal specificity. It promotes lipid synthesis by enhancing hepatic LXRα activity, thereby exacerbating inflammatory liver injury (7), while concurrently facilitating glycolysis in the tumor microenvironment through LDHA methylation (36). In immunomodulation, PRMT3 demonstrates a characteristic double-edged sword effect. On the one hand, it inhibits the production of type I interferon by methylating RNA and DNA sensors through the formation of complex modifiers, thereby compromising antiviral innate immunity. On the other hand, PRMT3-mediated inhibition of HSP60 methylation activates the cGAS/STING pathway, which enhances the anti-tumor immune response (37). Furthermore, PRMT3 contributes to tissue fibrosis and vascular calcification by modulating ADMA levels (8, 18) and also influences gestational immune tolerance. These multidimensional functions emphasize PRMT3’s crucial role in chronic inflammatory diseases while highlighting its dual potential as a therapeutic target. However, challenges such as tissue-specific effects, the molecular basis of substrate selectivity, and the complexity of cross-pathway interactions accompany this potential, all of which require systematic investigation. In subsequent sections, we will systematically delineate PRMT3’s regulatory network across metabolic, immune, and tissue damage pathways, while exploring its therapeutic translation potential.

### 4.1 PRMT3’s dual metabolic roles

#### 4.1.1 Lipid metabolism and inflammation

NAFLD, a metabolic disorder of escalating global incidence, is intricately linked to hepatic insulin resistance and aberrant lipogenesis (38). LXRα orchestrates hepatic lipid homeostasis through transcriptional regulation. Recent studies utilizing palmitic acid (PA)-treated cellular models have uncovered that hyperlipidemic stress induces widespread remodeling of protein arginine asymmetric dimethylation, concurrent with PRMT3 upregulation, lipogenic protein induction, and triglyceride accumulation (7). Functional analyses in HEK293 cells revealed that PRMT3 overexpression promotes lipogenesis via the LXRα signaling axis, manifested by augmented fatty acid biosynthesis and lipid droplet formation. Molecularly, PRMT3 acts as a transcriptional coactivator by binding to LXRα, thereby enhancing its chromatin occupancy and target gene expression. Notably, hepatic tissues from NAFLD patients exhibit elevated PRMT3 expression and increased LXRα-PRMT3 interaction.

**Figure 2:**
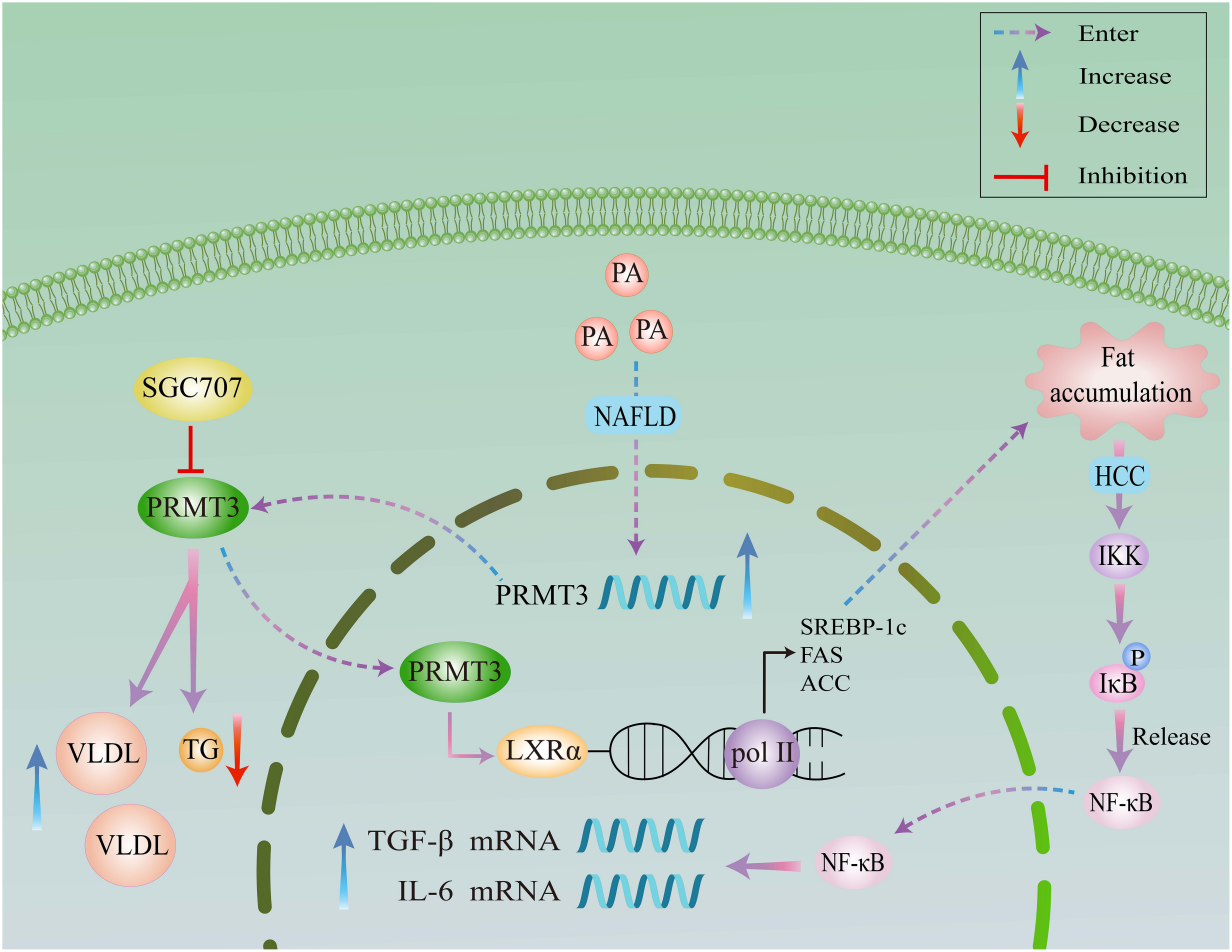
A schematic diagram of the molecular mechanism of PRMT3 in the occurrence and development of NAFLD and HCC. PRMT3 promotes lipidogenesis in NAFLD through the LXRα signaling axis, and promotes the progression of NAFLD to HCC via the NF-κB pathway. Meanwhile, it demonstrates the inhibitory effect of SGC707 on PRMT3 and the regulation of serum lipids.

Chronic lipid overload drives the progression from fatty liver to hepatocellular carcinoma (HCC) through induction of inflammatory responses, oxidative stress, and metabolic reprogramming, culminating in fibrosis, cirrhosis, and oncogenesis (39). Epidemiological data demonstrate that NAFLD patients face a dose-dependent increased risk of HCC development compared to non-NAFLD counterparts (40). Hyperlipidemic microenvironments activate macrophage inflammatory programs such as the NF-κB signaling pathway, indicating that PRMT3 may potentiate inflammatory responses through lipotoxicity-mediated mechanisms (41). However, while PRMT3 inhibitor SGC707 reduces serum lipids, it fails to alleviate atherosclerosis (42), indicating tissue-specific effects. Mechanistically, SGC707 decreases hepatic triglyceride content and VLDL secretion but paradoxically elevates hepatic cholesterol and plasma bile acids, concurrently inducing pruritus through undefined pathways (43), implicating neuroimmune regulatory roles (**Figure 2**).

#### 4.1.2 Glycolysis and inflammation

The glycolysis-inflammation axisacts as a central hub linking cellular metabolism and immune responses (44). Aberrant activation of the glycolysis-inflammation axis drives the progression of a wide range of diseases, forming a pro-cancer network through metabolic products, signaling pathways, and immunosuppressive cells, myeloid-derived suppressor cells (45). This process promotes tumor growth, metastasis, and treatment resistance. Metabolic enzymes such as glyceraldehyde 3-phosphate dehydrogenase and LDHA act as *in vivo* substrates of PRMT3, thus further regulating glycolysis. Moreover, studies have shown that PRMT3 is a critical regulator controlling the switch from oxidative phosphorylation to glycolysis in cancer cells, and cancer cells with high PRMT3 expression rely on glycolysis for their metabolic processes. (46).

In HCC, Lei et al. (36) established PRMT3 overexpression as an independent prognostic indicator. High PRMT3 levels correlate with advanced tumor stage and aggressive phenotypes, while genetic ablation of PRMT3 suppresses HCC cell proliferation and glycolytic flux. Mechanistically, PRMT3 methylates LDHA at arginine 112 through its catalytic E338 residue, augmenting LDHA enzymatic activity. This post-translational modification potentiates glycolysis, sustaining oncogenesis and lactate-mediated acidosis. The resulting tumor microenvironment promotes M2 macrophage polarization, underscoring PRMT3’s role in metabolic reprogramming and immune evasion. Despite its clinical utility, oxaliplatin resistance remains a major therapeutic obstacle. Shi et al. (17) identified PRMT3 as a determinant of chemoresistance, with high PRMT3 expression predicting poor outcomes. Proteomic analysis revealed IGF2BP1 as a novel PRMT3 substrate, where methylation at evolutionarily conserved arginine 452 is required for oxaliplatin resistance. Functional validation demonstrated that IGF2BP1 stabilizes HEG1 mRNA via m6A-dependent mechanisms, forming a PRMT3-IGF2BP1-HEG1 signaling axis. HEG1 knockdown abrogated both cell proliferation and drug resistance, whereas HEG1 overexpression rescued these phenotypes.

**Figure 3:**
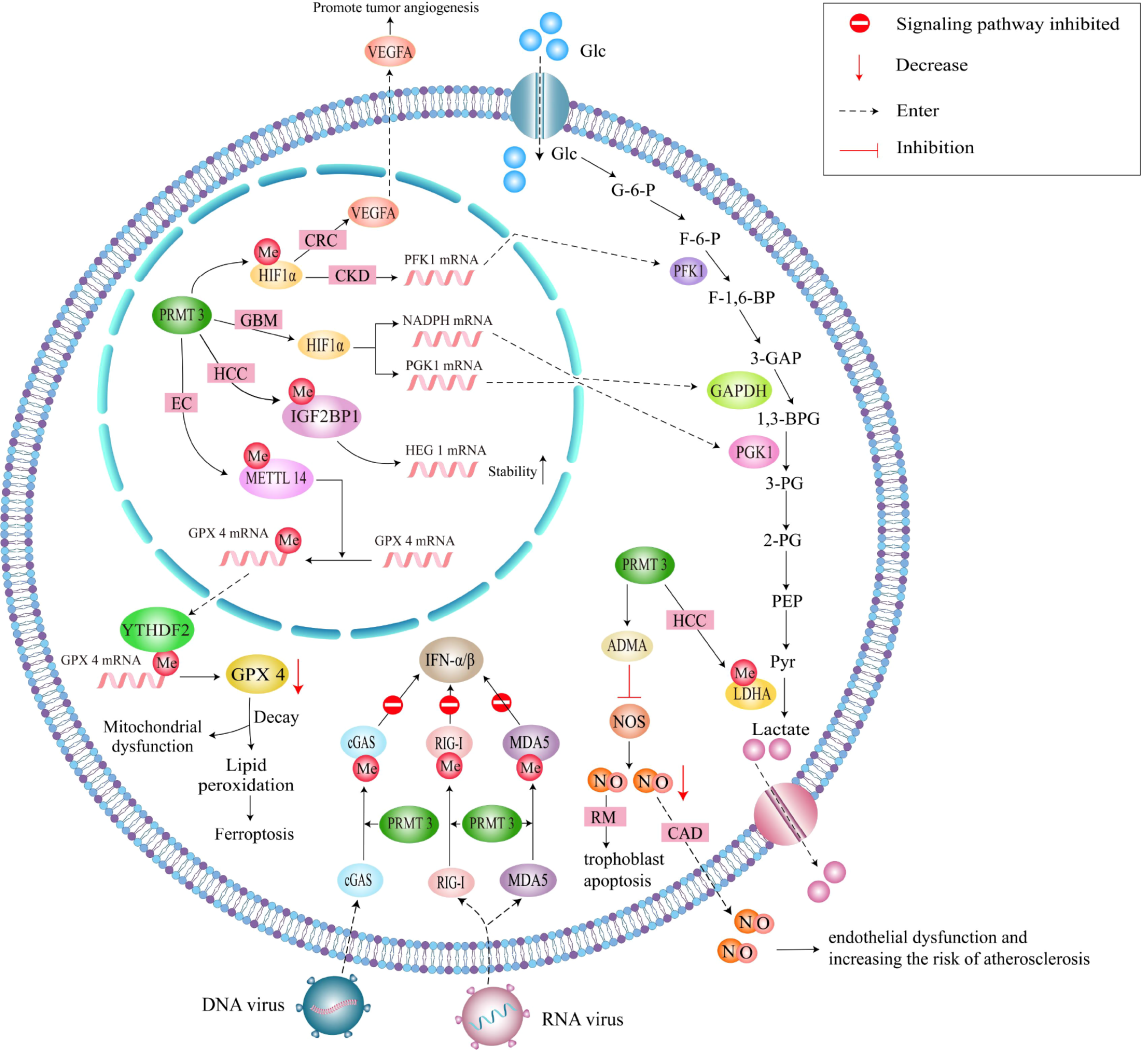
A schematic representation of the methylation-dependent regulatory pathways of PRMT3. This figure summarizes the PRMT3-methylated proteins that are involved in genetic and metabolic regulations.

In glioblastoma (GBM), PRMT3-mediated stabilization of HIF1α drives angiogenesis and glycolysis under hypoxic conditions (13). High PRMT3 expression correlates with glioma malignancy and poor prognosis, positioning it as a therapeutic target. In colorectal cancer, PRMT3 promotes tumorigenesis through dual mechanisms whereby it stabilizes HIF1α and METTL14 in a methylation-dependent manner, thereby enhancing glycolysis, angiogenesis, and inflammation (16, 17). Targeting the PRMT3-HIF1α axis through pharmacological inhibitors or methylation-blocking peptides may overcome anti-angiogenic resistance in colorectal cancer (**Figure 3**).

### 4.2 Dual roles of PRMT3 in immune regulation

#### 4.2.1 Suppression of antiviral innate immunity

The innate immune system serves as the first line of defense against viral pathogens, employing a variety of mechanisms to recognize and neutralize threats. Among these, the antiviral innate immunity is paramount for the detection of viral nucleic acids and the subsequent activation of immune responses. This initial immune response is critical for controlling viral infections and preventing disease progression. PRMT3’s role extends beyond target protein methylation to include regulation of key inflammatory pathways such as cGAS/STING and NF-κB. The cyclic cGAS and STING pathway are vital for sensing cytosolic DNA and initiating type I interferon responses. PRMT3 has been implicated in modulating this pathway by methylating proteins involved in cGAS/STING signaling, potentially influencing the activation of downstream interferon responses and pro-inflammatory signaling. By affecting this pathway, PRMT3 can amplify the immune response during infections or cellular stress, but it can also contribute to autoimmune inflammation when dysregulated. NF-κB is a key transcription factor in regulating immune responses and inflammation. PRMT3 can impact the NF-κB signaling pathway by methylating either NF-κB subunits directly or regulatory proteins that modulate its activity. The methylation status of these proteins can either promote or inhibit NF-κB activation, thereby influencing the expression of pro-inflammatory cytokines.

In addition to the cGAS/STING pathway, PRMT3 also modulates NF-κB signaling, a critical transcription factor that regulates inflammatory responses. The NF-κB pathway is activated in response to various stimuli, including pro-inflammatory cytokines and pathogen-associated molecular patterns. Upon activation, NF-κB translocates to the nucleus and drives the expression of numerous pro-inflammatory genes, which are essential for orchestrating an effective immune response. PRMT3 can influence NF-κB signaling by methylating its subunits or regulatory proteins that affect its activity. The methylation status of these proteins can either promote or inhibit NF-κB activation, thereby modulating the expression of key inflammatory cytokines such as TNF-α, IL-1β, and IL-6. Dysregulated NF-κB activity can lead to chronic inflammation, tissue damage, and contribute to the pathogenesis of autoimmune diseases (37). By negatively regulating this pathway, PRMT3 adds another layer of complexity to its role in modulating antiviral immunity. Given that NF-κB is a critical mediator of inflammation, PRMT3’s ability to regulate this pathway underscores its role in inflammatory diseases.

Antiviral innate immunity operates through the recognition of pathogenic nucleic acids by pattern recognition receptors. These receptors, including retinoic acid-inducible gene I (RIG-I) and cGAS, play crucial roles in detecting viral RNA and DNA, respectively. Upon recognition of viral components, these pattern recognition receptors initiate a cascade of signaling events that lead to the production of type I interferons and inflammatory cytokines. This cascade activates hundreds of antiviral effector proteins, including MX1 and OAS (47), which form a robust antiviral network that effectively counters viral replication and spread. The cGAS/STING pathway is critical for the detection of cytosolic DNA and the subsequent induction of interferon responses. When cGAS detects viral DNA within the cytoplasm, it catalyzes the synthesis of cyclic GMP-AMP, a second messenger that binds to and activates STING, an endoplasmic reticulum-localized protein. Activated STING translocates to the Golgi apparatus, leading to the recruitment of TANK-binding kinase 1 and the subsequent phosphorylation of interferon regulatory factor 3 (48). Phosphorylated interferon regulatory factor 3 dimerizes and translocates to the nucleus to initiate the transcription of type I interferons and other pro-inflammatory genes. This pathway is crucial for mounting an effective immune response to viral infections; however, the activation of the cGAS/STING pathway must be tightly regulated to prevent uncontrolled inflammation and tissue damage. Dysregulation of this signaling cascade can lead to chronic viral persistence and autoimmune diseases, marking the importance of mechanisms that finely tune antiviral responses.

Notably, the preferential modification of pattern recognition receptors by PRMT3 during viral infection is primarily driven by the coordinated regulation of substrate availability and the post-translational modification of PRMT3 itself. On one hand, viruses activate the Toll-like receptor/retinoic acid-inducible gene I-like receptor signaling pathway via pathogen-associated molecular patterns, which significantly induces the transcription and translation of RNA/DNA sensors such as cGAS and RIG-I. These sensors harbor abundant arginine-glycine domains in their molecular structures, which form stable hydrogen bonds with the hydrophobic pocket of PRMT3’s N-terminal C_2_H_2_ zinc finger domain. This designates the sensors as preferred substrates for PRMT3, allowing PRMT3 to bind them before metabolism-related substrates like LXRα or HIF-1α. On the other hand, virus-induced secretion of interferon-γ activates JAK1/2 kinases, which further phosphorylate PRMT3’s Tyr85 residue, increasing the zinc finger domain’s binding affinity for PRRs by approximately 2-fold. Meanwhile, viral infection also induces cellular expression of metallothioneins, which chelate cytoplasmic Zn²^+^ to restrict excessive PRMT3 activation. This forms a negative feedback loop in which PRMT3 inhibits antiviral signaling and MTs restrict PRMT3 activity, and this loop balances the intensity of immune suppression (49).

Antiviral innate immunity constitutes the host’s initial defense against viral pathogens. Pattern recognition receptors detect viral nucleic acids, triggering type I interferon (IFN-α/β) production and inflammatory cytokine release. RIG-I is instrumental in the recognition of viral RNA, and its activation leads to the induction of pro-inflammatory cytokines and type I interferons (50, 51). However, PRMT3-mediated methylation at R730 disrupts RIG-I oligomerization, a crucial step for its activation. When RIG-I fails to oligomerize properly, it is unable to effectively bind viral RNA, leading to a reduced interferon response. The consequence is a diminished antiviral state within the cell, facilitating viral replication and persistence. Similarly, MDA5, another key PRR that recognizes long double-stranded viral RNA, is also impacted by PRMT3 through methylation at R822. The perturbation of MDA5 activity through this modification interferes with its capability to form filamentous structures necessary for signal transduction, thereby attenuating the cytoplasmic signaling required for effective interferon production. This can contribute to viral immune evasion and may play a role in the pathogenesis of chronic viral infections. This activates hundreds of antiviral effector proteins including MX1 and OAS, which collectively form a broad-spectrum antiviral network. Key features include rapid activation, immune surveillance, and adjuvant roles in adaptive immunity via antigen presentation and co-stimulation. Dysregulation of this system contributes to chronic viral persistence, cytokine storms, and tumor immune evasion. Epigenetic regulators like PRMTs have emerged as critical nodes in antiviral defense, offering novel therapeutic opportunities.

cGAS serves as a sentinel for cytosolic DNA, and its activation leads to robust innate immune responses (52). By methylating cGAS at R111, PRMT3 inhibits its ability to bind DNA, thereby blunting the activation of the cGAS/STING pathway. This disruption not only dampens interferon production but also alters the expression of other pro-inflammatory mediators. The reduced activation of the cGAS/STING pathway means that the innate immune system’s ability to sense and respond to viral DNA is significantly impaired, thus fostering an environment conducive to viral persistence. PRMT3 negatively modulates antiviral responses by catalyzing asymmetric arginine dimethylation at R730 of RIG-I, R822 of MDA5, and R111 of cGAS (49). This post-translational modification disrupts receptor oligomerization and downstream interferon signaling, thereby attenuating nucleic acid binding and antiviral effector activation. This mechanism likely exacerbates chronic viral pathogenesis by blunting interferon responses, providing mechanistic insights into viral immune evasion and potential targets for therapeutic intervention.

#### 4.2.2 Promotion of pro-inflammatory signaling and immune evasion

PRMT3 plays a multifaceted role in regulating immune responses and maintaining mitochondrial integrity, and its dual functionality makes it a compelling subject for therapeutic exploration in cancer, particularly in immune evasion and pro-inflammatory signaling. One of the critical functions of PRMT3 lies in its ability to methylate HSP60 (53), a chaperone protein that is vital for maintaining mitochondrial function and integrity. Methylation of HSP60 by PRMT3 stabilizes its oligomeric state, thereby ensuring proper protein folding and facilitating mitochondrial protein quality control. This stabilization is essential not only for mitochondrial health but also for the immune response, particularly regarding the cGAS/STING pathway, which plays a vital role in sensing cytosolic DNA and initiating an antiviral response.

When PRMT3 methylates HSP60, it effectively suppresses the cGAS/STING activation pathway. This suppression can have significant implications in the context of cancer. Tumor cells often utilize mechanisms to evade immune detection, and the inhibition of the cGAS/STING pathway is one such strategy (54). By keeping this pathway in check, PRMT3 may allow tumors to escape immune surveillance, promoting an environment conducive to tumor growth and metastasis. Conversely, the application of PRMT3 inhibitors has been shown to reverse this suppression, leading to the activation of the cGAS/STING-dependent antitumor immunity. This crucial discovery positions PRMT3 not only as a biomarker for predicting responses to immunotherapy but also as a promising therapeutic target in the treatment of malignancies, such as hepatocellular carcinoma. Paradoxically, PRMT3 maintains mitochondrial integrity by methylating HSP60, which stabilizes its oligomeric state and suppresses cGAS/STING activation. PRMT3 inhibitors reverse this effect, activating cGAS/STING-dependent antitumor immunity (37). This dual functionality positions PRMT3 as a biomarker for predicting immunotherapy response and a therapeutic target in hepatocellular carcinoma.

In colorectal cancer, PRMT3 promotes immune evasion through c-MYC stabilization. By inhibiting c-MYC ubiquitination, PRMT3 enhances oncoprotein stability and downstream pro-tumorigenic signaling (55). These context-dependent roles highlight PRMT3’s functional plasticity, likely arising from differential substrate availability and post-translational modifications in distinct microenvironments (**Figure 3**). In the context of colorectal cancer, PRMT3’s role in promoting immune evasion becomes even more pronounced. One of the critical mechanisms is the stabilization of the oncogene c-MYC. c-MYC is a well-established transcription factor that regulates various cellular processes, including cell proliferation, metabolism, and apoptosis. In cancer, its overexpression is associated with increased tumor aggressiveness and poor patient outcomes (56). PRMT3 contributes to the stability of c-MYC by specifically inhibiting its ubiquitination, a post-translational modification that typically marks proteins for degradation by the proteasome. By preventing the ubiquitination of c-MYC, PRMT3 enhances the stability of this oncoprotein, leading to the persistent activation of downstream signaling pathways that favor tumorigenesis. These pathways often promote cell proliferation, enhance metabolic processes favoring tumor growth, and facilitate immune suppression. The interplay between PRMT3 and c-MYC exemplifies the complex regulatory networks in tumor microenvironments, where PRMT3 orchestrates pro-tumorigenic signaling. Additionally, c-MYC has been shown to regulate the expression of various immune checkpoints and suppress immune-related gene transcription, further facilitating immune evasion (57, 58). Thus, PRMT3’s role in stabilizing c-MYC creates a feedback loop that not only promotes tumor growth but also inhibits effective anti-tumor immune responses.

The preferential selection and efficient modification of oncogenic substrates by PRMT3 in the tumor microenvironment are mainly driven by the dual regulation of substrate availability and the post-translational modification of PRMT3 itself. From the substrate standpoint, either MYC activation or HIF-1α stabilization in tumor cells markedly upregulates the expression of oncogenic substrates such as HIF-1α and METTL14. The Arg residues of these substrates are surrounded by hydrophilic Ser/Thr amino acids, which form additional salt bridges with the Asp/Glu residues in PRMT3’s N-terminal zinc finger domain. Their binding affinity for PRMT3 is far greater than that of HSP60, enabling PRMT3 to preferentially modify oncogenic substrates (16). From the perspective of PRMT3 self-regulation, the abnormally activated PI3K-AKT pathway in tumors phosphorylates the Ser201 residue near PRMT3’s catalytic domain, directly enhancing its catalytic efficiency for substrates like HIF-1α and METTL14. Furthermore, p53 mutations in most tumors reduce the expression of its target gene MDM2, leading to decreased K48-linked ubiquitination of PRMT3. Consequently, PRMT3’s half-life is prolonged from 4 hours to 8 hours, providing an ample temporal window for its sustained modification of oncogenic substrates (14, 17).

The functional plasticity exhibited by PRMT3 underscores the importance of the tumor microenvironment in determining its roles and effects. This plasticity likely arises from differential substrate availability and variations in post-translational modifications that occur in response to specific microenvironmental signals. For instance, the availability of different target substrates may shift depending on the cellular context, whether it be in a pro-inflammatory or immunosuppressive microenvironment. In inflammatory conditions, cGAS/STING pathway activation can be beneficial for eliminating tumor cells, and PRMT3’s suppression of this pathway may represent a protective mechanism that cancer cells exploit. Conversely, in a microenvironment characterized by heightened immune pressure, PRMT3 may pivot toward enhancing immune evasion strategies through c-MYC stabilization or other tumor-promoting pathways. This duality illustrates that PRMT3’s influence is not static but rather responsive to external cues within the tumor microenvironment.

### 4.3 PRMT3 regulation in tissue injury and fibrosis

#### 4.3.1 Molecular basis of PRMT3-mediated regulation of ADMA-NO axis

PRMT3 regulates tissue injury and fibrosis primarily through mediating the ADMA-NO axis signaling pathway. PRMT3 catalyzes the conversion of substrate arginine to ADMA in two sequential steps, relying on the dual E-loop and His254/Asp256 proton transfer system within its C-terminal catalytic domain. It firstly converts arginine to MMA, followed by further conversion of MMA to ADMA. Additionally, its N-terminal C_2_H_2_ zinc finger domain preferentially recognizes ADMA precursor proteins containing the “Arg-Gly-Gly motif”, ensuring substrate specificity for ADMA synthesis.

This pathway exhibits a positive feedback loop of ADMA→PRMT3→ADMA. ADMA upregulates the transcriptional and protein expression of PRMT3, which in turn further promotes ADMA synthesis, forming a vicious cycle that exacerbates NO inhibition (59). ADMA inhibits NO through two distinct mechanisms. Firstly, it competitively binds to the active site of NOS, directly blocking NOS-mediated conversion of L-arginine to NO. Secondly, it induces NOS uncoupling, which prevents NOS from generating NO normally and instead triggers the production of ROS. Ultimately, this leads to reduced NO bioavailability and the loss of NO’s tissue-protective effects (42, 60).

#### 4.3.2 Renal and hepatic fibrosis

CKD affecting 10%-13% of the global population (61), poses a significant public health challenge. Tubulointerstitial fibrosis represents the common pathological pathway for diverse renal disorders progressing to end-stage kidney disease. PRMTs epigenetic regulators critical for cellular homeostasis, have been implicated in renal protection through type I PRMTs. PRMT3, a type I PRMT, plays a key role in ADMA biosynthesis. ADMA, primarily cleared by renal excretion, accumulates in chronic renal failure patients (62), where plasma levels correlate with disease progression. Mechanistically, PRMT3-mediated ADMA accumulation alleviates tubulointerstitial fibrosis (18). Notably, ADMA serves as a biomarker for endothelial dysfunction and inflammation. In coronary artery disease (60), PRMT3 expression positively correlates with myocardial ADMA levels. Elevated ADMA in coronary endothelial cells upregulates PRMT3 expression, which inhibits nitric oxide synthase, thereby increasing atherosclerotic risk (63).

Animal models and clinical evidence further validate the role of the PRMT3-ADMA axis in renal fibrosis. In a mouse model of unilateral ureteral obstruction, PRMT3 mRNA and protein levels were significantly upregulated in the obstructed kidneys. In contrast, following PRMT3 gene knockout, renal tissue ADMA levels decreased rather than increased, accompanied by a significant rise in collagen deposition. Exogenous injection of ADMA reversed the aggravated fibrotic phenotype in PRMT3-knockout mice, directly confirming that ADMA acts as a downstream effector molecule of PRMT3 in suppressing renal fibrosis (18). In clinical samples, PRMT3 expression in renal tissues of patients with CKD was positively correlated with ADMA levels. Moreover, as a well-recognized marker of endothelial dysfunction, ADMA reduces NO production by inhibiting NOS activity (64). This moderate NO inhibition effect prevents excessive NO-mediated renal tubular injury and exacerbated fibrosis, providing a rational basis for targeting this axis in anti-fibrotic therapy for CKD (65).

#### 4.3.3 Vascular calcification and pregnancy complications

CKD is characterized by irreversible progression and cardiovascular complications, with vascular calcification emerging as a critical mediator (66). Osteoblastic transdifferentiation of VSMCs underlies this process, which is linked to glycolytic reprogramming (67). HIF-1α is a critical transcription factor that responds to cellular oxygen levels and plays a crucial role in the adaptation to hypoxia (68). Under inflammatory conditions, PRMT3 has been shown to methylate HIF-1α, which enhances its stability and transcriptional activity. When HIF-1α is methylated, it promotes the expression of genes involved in angiogenesis, glucose metabolism, and pro-inflammatory cytokine production. This methylation process is crucial during inflammation, as it not only helps cells adapt to low oxygen conditions but also perpetuates the inflammatory response. Zhou et al. (8)demonstrated PRMT3 upregulation in CKD-affected medial VSMCs and β-glycerophosphate-induced calcification models. PRMT3 promotes glycolysis through HIF-1α methylation, exacerbating CKD-associated vascular calcification and inflammatory responses.

In recurrent miscarriage (RM) models, macrophage PRMT3 downregulation reduces NO bioavailability, inducing trophoblast apoptosis and perturbing maternal-fetal interface function, suggesting PRMT3 involvement in pregnancy-related inflammation (**Figure 3**). LXRa is a nuclear receptor that regulates lipid metabolism and inflammation. PRMT3 mediates the methylation of LXRa, affecting its transcriptional activity. Methylation of LXRa can modulate the receptor’s interaction with coactivators and corepressors, thus impacting its ability to regulate genes involved in lipid homeostasis and inflammation. In the context of inflammatory diseases, the altered function of LXRa due to PRMT3 activity can contribute to dysregulated lipid metabolism, which is often associated with chronic inflammation. ADMA is an endogenous inhibitor of nitric oxide synthase, which plays a vital role in maintaining vascular health. PRMT3 alters the levels of ADMA by methylating specific proteins involved in its synthesis and degradation. Elevated levels of ADMA can lead to reduced nitric oxide production, promoting vascular dysfunction and inflammation. By modulating ADMA levels, PRMT3 influences nitric oxide signaling pathways that are crucial for maintaining vascular tone and inflammatory responses.

In RM, abnormal activation of the PRMT3-ADMA-NO axis represents a key pathological mechanism. PRMT3 is specifically overexpressed in decidual macrophages of RM patients, where it catalyzes ADMA production by acting on substrates containing the RGG motif. ADMA then competitively binds to the active site of NOS, significantly reducing NO synthesis in decidual tissue. NO deficiency subsequently activates the Fas/FasL pathway, inducing the activation of Caspase-3/7/9 and ultimately leading to trophoblast cell apoptosis, which disrupts the homeostasis of the maternal-fetal interface. Functional validation showed that SGC707, a PRMT3 inhibitor, reduced ADMA levels in macrophages of RM model mice, restored NO concentrations, and decreased embryo resorption rates (11). These findings confirm that targeting the PRMT3-ADMA-NO axis can improve pregnancy outcomes, offering a novel direction for RM treatment.

### 4.4 Impact of PRMT3 activity on autoimmune diseases

Unlike cGAS activation by exogenous nucleic acids during viral infection, autoimmune diseases are characterized by self-nucleic acid leakage. For instance, self-DNA released by neutrophil extracellular traps and cytoplasmic double-stranded DNA from uncleared apoptotic cells (69). These self-nucleic acids persistently activate the cGAS/STING pathway, triggering a type I interferon storm and excessive secretion of proinflammatory cytokines, ultimately contributing to chronic autoimmune inflammation.

Under normal physiological conditions, PRMT3 maintains the low-activity homeostasis of the cGAS/STING pathway by methylating Arg111 of cGAS to prevent its binding to DNA and by inhibiting the dimerization of STING. However, when PRMT3 expression is downregulated or its function is compromised, its inhibitory effect on the cGAS/STING pathway is abrogated, resulting in pathway hyperactivation and subsequently inducing autoimmune responses. Specifically, the existing literature (70) indicates that abnormal PRMT3 activity is likely to contribute to RA pathogenesis and progression via the following mechanism. RA patients often present with iron deficiency anemia, which in turn downregulates PRMT3 expression and activity. As PRMT3 acts as a negative regulator of the cGAS/STING pathway, reduced PRMT3 diminishes inhibition of the pathway, thereby driving its hyperactivation. This hyperactivation further promotes sustained release of type I interferons and proinflammatory cytokines, which exacerbates chronic inflammation and worsens rheumatoid arthritis-associated pathological features, including synovial inflammation and joint damage.

## 5 Therapeutic potential and challenges of PRMT3 inhibitors

**Figure 4:**
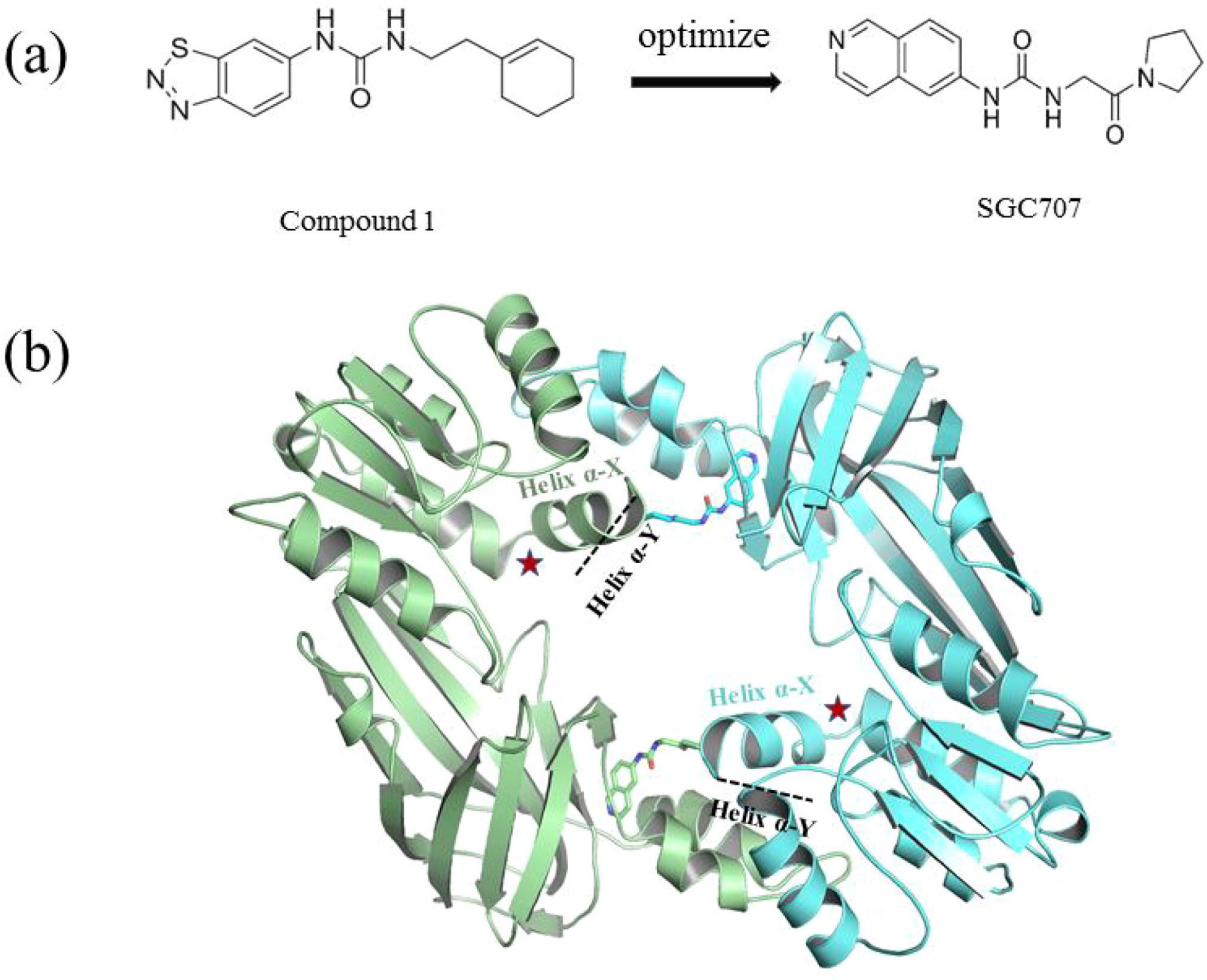
**(a)** Discovery of Compound 1 and SGC707. **(b)** Canonical dimerization of PRMT3 (PDB: 4RYL). The labeled regions helix α-X and α-Y are two conserved α-helical domains of PRMT3. Among them, the helix α-X is a critical dynamic helix located at the interface of the β-barrel and dimerization arm, and is essential for maintaining the catalytic activity of PRMT3. The red star indicates the site of methyl transfer. .

PRMT3 inhibitors represent promising therapeutic candidates for cancer and metabolic disorders (**Table 1**). The first allosteric inhibitor, Compound 1, was identified in 2012 via high-throughput screening of 16,000 compounds (5). Subsequent optimization yielded SGC707 (71), which demonstrated enhanced potency and selectivity while showing no detectable activity against 31 methyltransferases or over 250 non-epigenetic targets in comprehensive profiling (**Figure 4a**). SGC707 binds at the β-barrel/dimerization arm interface (PDB: 4RYL), destabilizing the α-X helix critical for catalytic function (5, 71) (**Figure 4b**). This dynamic helix is conserved across class I PRMTs. In cellular models, SGC707 stabilizes PRMT3, reduces H4R3me2a histone marks, and exhibits favorable pharmacokinetic profiles.

**Table 1:** The classification, functions and significance of PRMT3 inhibitors.

PRMT3 inhibitors	Structure diagram	Type	PRMT3 IC_50_	K_D_	Biological function	Clinical significance	References
Compound 1	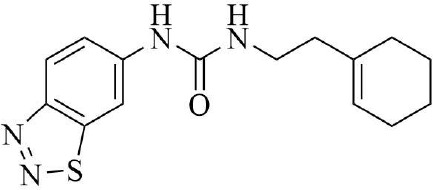	Allosteric Inhibitors	2.5 ± 0.1 μM 1.6 ± 0.3 μM 2 ± 0.5 μM	9.5 ± 0.5 μM	• It binds to the allosteric pocket of PRMT3 and exerts an inhibitory effect.	• Novel PRMT3 inhibitors can be designed based on their specific inhibitory effects.	(5)
					• Its inhibition of PRMT3 activity selectively suppresses LXR-driven transcription of hepatic lipogenic genes, thereby reducing lipid synthesis.	• Its combination therapy targeting PRMT3 and the LXR pathway inhibits hepatic lipid synthesis without affecting the ability of LXR agonists to stimulate cholesterol efflux from macrophages.	(43)
SGC707	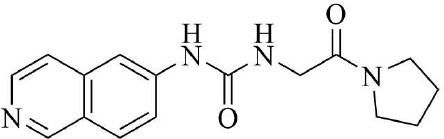	Allosteric Inhibitors	31 ± 2 nM	53 ± 2 nM	• In hepatocellular carcinoma, SGC707 can inhibit the activity of PRMT3 and affect the methylation of LDHA by PRMT3; It can also block the enhancement of glycolysis induced by the overexpression of PRMT3.	• SGC707 demonstrated the ability to inhibit glycolysis and tumor growth in hepatocellular carcinoma cells, providing a potential new target and therapeutic strategy for hepatocellular carcinoma treatment.	(36)
					• In non-alcoholic fatty liver disease and atherosclerosis, SGC707 inhibits PRMT3 activity, reduces hepatic triglyceride accumulation, affects white adipocyte size, alters plasma bile acid levels and TGR5 activation.	• By inhibiting PRMT3 activity, SGC707 reduces hepatic triglyceride accumulation and lowers plasma triglyceride levels, thereby ameliorating hepatic steatosis and dyslipidemia.	(43)
SGC707	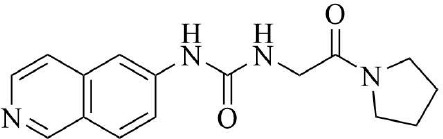	Allosteric Inhibitors	31 ± 2 nM	53 ± 2 nM	• SGC707 can inhibit GBMcell growth, inhibit cell migration and regulate expression of related proteins.	• SGC707 inhibits the growth of GBM cells by suppressing the function of PRMT3. Therefore, PRMT3 may become an important therapeutic target for GBM.	(13)
					• SGC707 inhibits the activity of PRMT3, which enhances the sensitivity of endometrial cancer cells to iron death.	• Combining SGC707 with anti-PD-1 therapy, chemotherapy, or radiotherapy can enhance treatment efficacy.	(17)
					• SGC707 inhibits the methyltransferase activity of PRMT3 and thus enhances the antiviral innate immune response.	• SGC707 can provide potential drug targets for the treatment of viral infectious diseases.	(49)
					• SGC707 can significantly attenuates vascular calcification, inhibits glycolysis, and regulates HIF-1α methylation in CKD mice.	• The ability of SGC707 to reduce vascular calcification and inhibit glycolysis suggests its potential as a therapeutic agent to improve vascular calcification in CKD patients.	(8)
SGC707	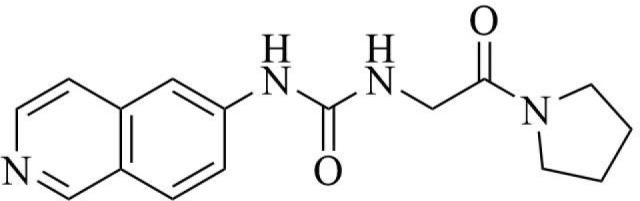	Allosteric Inhibitors	31 ± 2 nM	53 ± 2 nM	• SGC70 can decrease ADMA accumulation and increased NO concentration in macrophages, inhibited trophoblast apoptosis, and decreased embryo resorption in mice.	• Its interventions targeting the PRMT3/ADMA/NO pathway, such as the use of SGC707 to inhibit PRMT3 activity, may be a novel strategy for the treatment of RM.	(11)
					• SGC707 inhibits the methyltransferase activity of PRMT3, which in turn reduces the expression level of H4R3me2a, and induces a decrease in the amount of bone in mice.	• The inhibitory effect of SGC707 on PRMT3 suggests its potential use in regulating bone metabolism, providing a potential target for pharmacological intervention in the treatment of bone metabolic disease.	(21)
XY 1	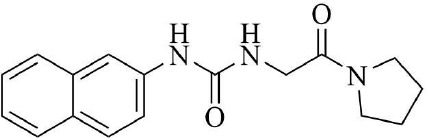	Negative control compound	–	–	• XYI contains a naphthalene group that replaces the isoquinoline group in SGC707 and has no inhibitory activity against PRMT3.	• XY1 is in sharp contrast to SGC707, which helps to study the action characteristics of SGC707.	(71)
Proteolysis Targeting Chimeras (PROTACs) 11	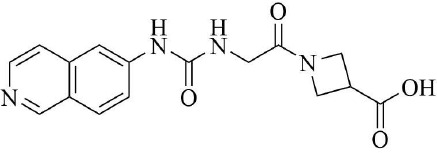	Degrader	–	–	• PROTACs11 inhibits cell growth, reduces ADMA levels and modulates related signalling pathways in multiple acute leukaemia cell lines.	• PROTACs11 showed more pronounced efficacy than conventional PRMT3 inhibitors in the treatment of acute leukaemia, providing a new potential strategy and drug option for the treatment of acute leukaemia.	(14)
Compound 14u	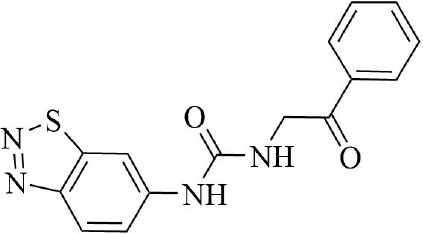	Allosteric Inhibitors	0.48 ± 0.01 μM	–	• It inhibits the activity of PRMT3 and reduces its methylation of substrates.	• It has emerged as a potential lead compound for research into the treatment of PRMT3-related diseases.	(74)
Compound 29	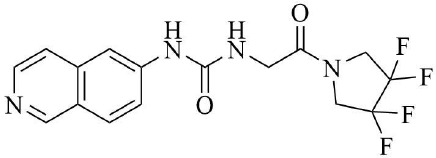	Allosteric Inhibitors	24 ± 3nM	–	• They bind to PRMT3 and effectively inhibit its methyltransferase activity, i.e. inhibit the asymmetric dimethylation of H4R3, which may affect PRMT3-related biological processes such as ribosome biosynthesis and cellular metabolism.	• By inhibiting PRMT3 activity, they may provide new therapeutic strategies and potential drug targets for the treatment of diseases associated with PRMT3 abnormalities, such as cardiovascular diseases and tumours.	(75)
Compound 30	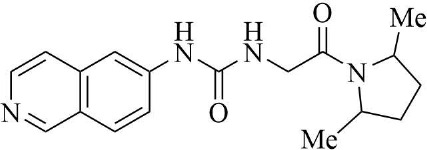		22 ± 2 nM				
Compound 36	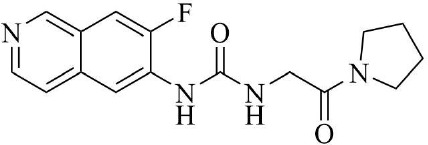		19 ± 4 nM				

These inhibitors modulate lipid metabolism through LXRα signaling while concurrently targeting tumor glycolysis via HIF1α regulation. In 2015, Kaniskan et al. (71) engineered SGC707 with improved cellular activity, which suppressed glioblastoma xenograft growth and showed preclinical efficacy. Studies (42) in hyperlipidemic apoE knockout mice revealed SGC707 reduced hepatic steatosis and triglyceride accumulation, coinciding with adipose tissue remodeling. Recent findings (17) highlight SGC707’s potential to enhance endometrial cancer immunotherapy through lipid peroxidation induction and GPX4 downregulation. However, side effects and target selectivity require refinement (43). These inhibitors modulate lipid metabolism through LXRα signaling while concurrently targeting tumor glycolysis via HIF1α regulation. For example, targeting the PRMT3-HSP60 axis activates the cGAS/STING pathway to augment immunotherapy (37), yet avoids crosstalk with the LXRα pathway. Future development should integrate structural biology and disease modeling to advance next-generation inhibitors and combination strategies.

## 6 Conclusions and perspectives

PRMT3 functions as a pleiotropic methyltransferase generating ADMA, orchestrating inflammation through metabolic reprogramming, immune regulation, and epigenetic mechanisms. The methylation status of PRMT3 exhibits mechanistic associations with pathological processes. Iron deficiency (70) synergizes with DAL-1/4.1B tumor suppressor interactions to downregulate PRMT3’s enzymatic activity, whereas ALDH1A1 complex formation suppresses retinoic acid signaling pathways (72). These opposing regulatory mechanisms collectively govern cellular differentiation and proliferative capacity. Current evidence establishes (14) PRMT3 as a validated therapeutic target, as demonstrated by the emerging therapeutic efficacy of proteolysis-targeting chimeras (PROTACs) (73) in preclinical models of leukemia. In metabolic-inflammatory axes, PRMT3 drives lipid accumulation and glycolysis via LXRα, LDHA, and HIF1α, fostering pro-inflammatory microenvironments. PRMT3-mediated immune regulation manifests a context-dependent duality, with potent antiviral suppression contrasting its capacity to enhance antitumor responses. The tissue-specific regulatory functions of PRMT3 introduce mechanistic complexity through paradoxical attenuation of renal fibrosis concurrent with promotion of pancreatobiliary fibrotic progression and neoplastic transformation.

However, several critical limitations remain. Firstly, current research methodologies are constrained by reliance on gene knockout models and broad-spectrum inhibitors, hindering precise identification of enzymatic substrates. The cell-type-dependent regulatory roles of PRMT3 in immune systems remain insufficiently characterized, necessitating advanced approaches such as single-cell resolution sequencing and tissue-specific conditional knockout models. Secondly, translational challenges persist between preclinical and clinical stages, with limited dynamic validation in human-derived tissue systems. Current PRMT3 inhibitors face clinical limitations due to off-target effects, highlighting the demand for innovative delivery strategies including nanocarrier systems and proteolysis-targeting chimeras to achieve tissue-selective therapeutic targeting. Thirdly, synergistic interactions between PRMT3 and other PRMT family members such as PRMT1 remain poorly defined. Emerging connections to nitric oxide metabolic pathways and iron regulatory networks present novel opportunities for modulating inflammatory pathologies. Future research must prioritize three key areas. Developing spatiotemporally controlled PRMT3 inhibitors, elucidating inflammasome interactions and validating ADMA as a prognostic biomarker in human cohorts.
